# The Effect of Brain-Derived Neurotrophic Factor on Periodontal Furcation Defects

**DOI:** 10.1371/journal.pone.0084845

**Published:** 2014-01-14

**Authors:** Ryo Jimbo, Nick Tovar, Malvin N. Janal, Ramy Mousa, Charles Marin, Daniel Yoo, Hellen S. Teixeira, Rodolfo B. Anchieta, Estevam A. Bonfante, Akihiro Konishi, Katsuhiro Takeda, Hidemi Kurihara, Paulo G. Coelho

**Affiliations:** 1 Department of Prosthodontics, Faculty of Odontology, Malmo University, Malmö, Sweden; 2 Department of Biomaterials and Biomimetics, New York University College of Dentistry, New York, New York, United States of America; 3 Department of Epidemiology and Health Promotion, New York University, New York, United States of America; 4 Department of Dentistry, UNIGRANRIO, Duque de Caxias, Rio de Janeiro, Brazil; 5 Department of Prosthodontics, Integrated Center for Research, Bauru School of Dentistry, University of São Paulo, Bauru, São Paulo, Brazil; 6 Department of Prosthodontics, Integrated Center for Research, Bauru School of Dentistry, University of São Paulo, Bauru, São Paulo, Brazil; 7 Department of Periodontal Medicine, Division of Applied Life Sciences, Hiroshima University Institute of Biomedical & Health Sciences, Minami-ku, Hiroshima, Japan; University of North Carolina at Chapel Hill, United States of America

## Abstract

This study aimed to observe the regenerative effect of brain-derived neurotrophic factor (BDNF) in a non-human primate furcation defect model. Class II furcation defects were created in the first and second molars of 8 non-human primates to simulate a clinical situation. The defect was filled with either, Group A: BDNF (500 µg/ml) in high-molecular weight-hyaluronic acid (HMW-HA), Group B: BDNF (50 µg/ml) in HMW-HA, Group C: HMW-HA acid only, Group D: empty defect, or Group E: BDNF (500 µg/ml) in saline. The healing status for all groups was observed at different time-points with micro computed tomography. The animals were euthanized after 11 weeks, and the tooth-bone specimens were subjected to histologic processing. The results showed that all groups seemed to successfully regenerate the alveolar buccal bone, however, only Group A regenerated the entire periodontal tissue, *i.e.*, alveolar bone, cementum and periodontal ligament. It is suggested that the use of BDNF in combination with a scaffold such as the hyaluronic acid in periodontal furcation defects may be an effective treatment option.

## Introduction

Brain derived neurotrophic factor (BDNF), a second member of the neurotrophin family, is involved in the survival and differentiation of central and peripheral neurons [1] by directly affecting them to prevent apoptosis [2]. Furthermore, the presence of BDNF is essential for the expression of long-term potentiation in the hippocampus [3], which indicates that the factor is involved in the synaptic plasticity formation that fulfills many of the criteria for a neural correlation of memory [4]. Due to its specific role during neurotransmission, the balance of BDNF has been reported to provoke neurological and psychiatric disorders [5], since the expression level of BDNF mRNA significantly decreases under stress induction [6].

In addition, previous studies have shown that BNDF is also expressed by a variety of non-neural cells and tissues outside of the nervous system [7]. BDNF also has an effect on odontoblast differentiation [8] and bone remodeling [9] by binding to appropriate high-affinity receptors such as the receptor tyrosine kinase (Trk). Thus, the multiple roles played by BDNF in a multitude of tissues and at different scenarios have rendered BDNF a candidate for in tandem multi tissue regeneration in complex clinical scenarios.

Concerning periodontal tissue regeneration, BDNF reportedly increased the synthesis of osteopontin, BMP-2, and collagen in human periodontal ligament (PDL) cells [10], strongly suggesting the existence of the Trk signaling pathway, which the receptor selectively binds to the BDNF [11]. It was further confirmed that a specific signaling pathway, TrkB-c-Raf-ERK1/2-Elk-1 was essential for the BDNF to induce mRNA expression of bone/cementum-related proteins [12] and also for the regulation of cementoblast survival [13]. *In vivo* application of this promising neurotrophic factor in a canine Class III furcation defect showed that BDNF can regenerate new alveolar bone, cementum, and connective fibers attached to the newly formed cementum [10], [14]. One of the above studies reported that BDNF immersed into a commercially available bovine atelocollagen sponge significantly regenerated periodontal tissues compared to the control, where only the atelocollagen sponge was inserted [10].

In another study, a synthesized high-molecular weight-hyaluronic acid (HMW-HA) was utilized as a scaffold for BDNF, which presented high levels of periodontal tissue regeneration indicating the synergistic effect of the HMW-HA and the BDNF [14]. Since the periodontal tissue regeneration of a furcation defect is one of the most challenging cases in regenerative dentistry, the clinical application of the BDNF-scaffold is of great interest. However, extrapolating these experimental results to clinical practice is a challenge and further investigation considering a hierarchically higher laboratory *in vivo* model is desirable for determining the levels of BDNF application that are clinically relevant.

Therefore, the current study investigated different dosages of BDNF when combined with HMW-HA for periodontal tissue regeneration in a non-human primate model.

## Materials and Methods

### Laboratory Animal Model

This study consisted of eight healthy non-human primate (NHP) individuals, *Macaca Fascicularis* (approximately 8 years of age), and was performed under approval of the committee for animal experimentation at the National Veterinary School of Lyon, France.

The animals were housed in groups of 2 or 3 in stainless-steel cages under a 12D-12N lightning cycle at a room temperature of 22°C +/−3°C (ventilation: 10 air changes/hour, with no air recirculation). Animal room and cage cleaning was performed daily. Specific primate diet (SDS OWM) was provided daily in appropriate amounts for the size and age of the animals (100 g for animals under 5 kg and 200 g for animals over 5 kg). Delicacies were also occasionally given to the animals as part of the Testing Facility environmental enrichment program. Tap water was available ad libitum to each animal via an automatic watering device.

The study animals were acclimated to their designated housing for at least 21 days prior to the first day of the study.

For a pre-study screening, a complete examination, under ketamine sedation, was conducted by a veterinarian, which included abdominal palpation, observations of the condition of integument, and respiratory and cardiovascular systems.

All surgical procedures were performed under general anesthesia. As premedication, 10 mg/kg of ketamine (Ketamine 1000®, Virbac, France) and 1 mg/kg of midazolam (Midazolam® 5 mg/ml, Aguettant, France) were injected intramuscularly (IM). Stress during injection was limited as much as possible.

**Figure 1:**
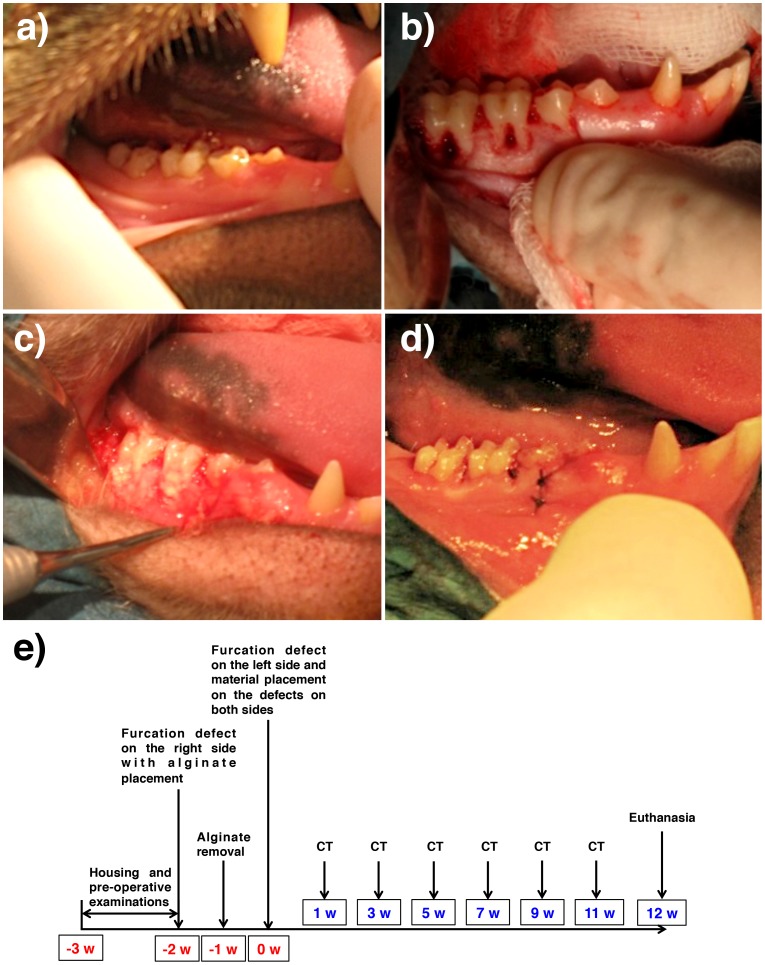
Design of the experiment. (a) Pre operative condition of the oral cavity. All animals used were in periodontally healthy condition. (b) Full removal of the buccal plate and defect creation at the furcation region, (c) alginate placement to induce inflammation, and (d) suture. (e) The schematic timeline of the current study.

For all first and second molars involved in the present study (Figure 1a), the surgical procedure followed a standard periodontal techniques; following an intrasulcular incision, mucoperiosteal flaps were reflected and the bone tissue exposed at both buccal and lingual/palatal flanges of each presented 5 mm in height from the furcation level around each tooth (class II furcation lesion following the classification proposed by Lindhe and Nyman [15]). A furcation defect of 3 mm in depth was also created for each molar (Figure 1b). The defects were created by means of a 2 mm diameter cylindrical bur, and the periodontal ligament and cementum were smoothened by a hand scaler to produce denuded root surfaces (Figure 1b).

Approximately 14 weeks prior to sacrifice, the first surgical procedure was carried out on the right first and second molars of both jaws. After defect creation, alginate impression material was placed in the defects to induce experimental inflammation (Figure 1c) following the protocol used in out previous publication [14]. Seven days following defect creation on the right side, the sites were reopened; alginate removed and allowed to heal for 7 days. At this time frame, the left side first and second molars were subjected to the same defect creation procedure, and both the right and left side defects were then allocated to five different experimental groups. Such distribution allowed observations between sites with and without induced inflammation.

Five experimental groups were considered:

Group A: BDNF (500 µg/ml) in high molecular weight hyaluronic acid (HMW-HA, DENKA Co., Ltd., Tokyo, Japan)
Group B: BDNF (50 µg/ml) in HMW-HA
Group C: HMW-HA only
Group D: empty defect, control
Group E: BDNF (500 µg/ml) in saline solution

The defects on the first four NHPs were filled in an interpolated fashion with groups A, B, C, and D, whereas the defects on the last four NHPs were filled with materials A, B, C, and E.

The experimental group and site distribution were changed for each subject and resulted in a balanced study design (group per site). Following placement of the different materials, the flaps were coronally repositioned with resorbable sutures (Figure 1d, 4-0 Vicryl, Ethicon Inc., Somerville, NJ, USA). After surgery, the animals were subjected to a soft diet in order to prevent food physical interference with the surgical sites. After 12 weeks, the euthanasia was performed by anesthesia overdose by Ketamine 1000® (10 mg/kg) and terminated by intravenous injection of 10 mL Dolethal® at 200 mg/mL and exsanguination. The schematic diagram, which shows the time schedule of the study, is presented in Figure 1e.

### Computed Tomography Scans and Bone Defect Healing Quantification

Computed tomography (CT) scans were performed at the day the defects were filled with the experimental groups, and at approximately days 27, 42, 56, 70, 84 and 98 after first surgical procedure, which corresponded to 1, 3, 5, 7, 9, and 11 weeks following defect filling with the materials on both sides. A GE NXI Pro (GE Medical Systems, Madison, WI, USA) was used at an energy level of 120 kV and a current of 200 mA (focus volume, 10 cm in diameter).

### Histological processing and histomorphometry

Following CT, the specimens were fixed in 10% buffered formalin and reduced to four blocks containing one tooth each. After dehydration and infiltration in methacrylate-based resin, 30 µm thickness non-decalcified sections were obtained per block. Three sections were generated in the buccal lingual direction from the cervical to apical direction within the defect and referred to light microscopy evaluation. The patterns of new bone, cementum, and (PDL) were categorized as follows for each section:

- Bone: 1- full bone regeneration within defect, 2- partial bone regeneration within defect regions, and 3- no bone regeneration within defect.
- Cementum: 1- full cementum coverage of exposed dentin, 2- partial cementum coverage of exposed dentin, and 3- no cementum coverage of exposed dentin. Concerning cementum morphology: 1- cellular cementum, 2- acellular cementum, 3- mixed (cellular and acellular component), and 4- no cementum.
- PDL: 1- full fibrous content bridging bone and cementum, 2- partial fibrous content bridging bone and cementum, and 3- no fibrous bridging bone and cementum.

### Statistical Analysis

Statistical evaluation of bone formation through µCT scan quantification was performed by a mixed model ANOVA with fixed factors of treatment, time *in vivo*, arch and induced inflammation and a random intercept. For the histological data, we described three outcomes, full, partial, or no regeneration of bone cementum and PDL layers, but analyzed only full vs. non-full regeneration. Partial and no regeneration levels were aggregated for statistical analysis because the prevalence of each outcome (in at least some analyses) was too small to be considered on their own. Because each animal allowed 8 treatment sites, treatments A, B, and C was tested in all animals, while treatments D and E was each tested in half of the animals (with and without the inflammatory challenge). The histological analysis considered data from 13 teeth in treatment A, 16 in B, 15 in C, and 7 in each of D and E, with and without induced inflammation. Generalized estimating equations were then used with a logistic link to estimate effects of group and inflammation. This approach is conceptually similar to logistic regression but optimized for correlated observations. In all analyses, post-hoc comparisons were based on estimated confidence limits using the pooled estimate of the standard error for the mixed model ANOVA and Wald estimates for the GEE model. All statistical analysis used IBM SPSS v 21 (Armonk, NY).

## Results

**Figure 2:**
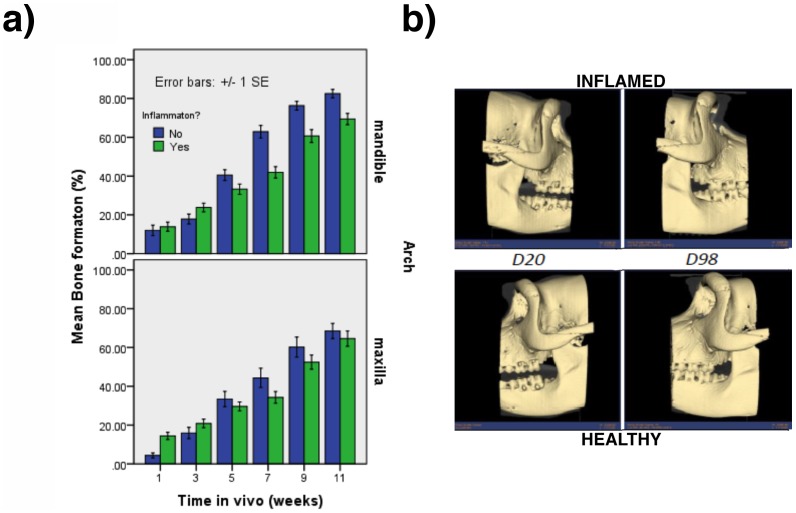
The comparison between induced inflammation versus non-inflamed sites. (a) Graph representing the effect of interactions between Arch, Inflammation, and Time. (b) Representative 3D reconstructed image of the furcation defects healing overtime for both inflamed (top) and healthy (bottom) sites. No differences were seen in furcation defect healing for both conditions.

Overall, no complications in either trans-op or post-op times were detected in any of the NHPs. The CT scan based quantification showed interactions between arch and induced inflammation (p = .01), arch and time (p = .04) and induced inflammation and time (p<.001). The effects are illustrated in Figure 2a along with reconstructed 3 dimensional images (Figure 2b). One sees, first, that the presence of induced inflammation interfered with bone formation more in the mandible than the maxillary arches; second, that bone formation proceeded more slowly in the maxillary than the mandibular arches; and third, that induced inflammation interfered with bone formation only at later healing times. In general, there were no differences in bone formation among treatment groups (p = .22) but treatment did interact with arch (p<.001), such that there were similarly low levels of bone formation for each treatment in the maxillary locations, but higher levels bone formation for treatments A, D and E than for treatment B and C in the mandibular arch (p<. 05).

**Figure 3:**
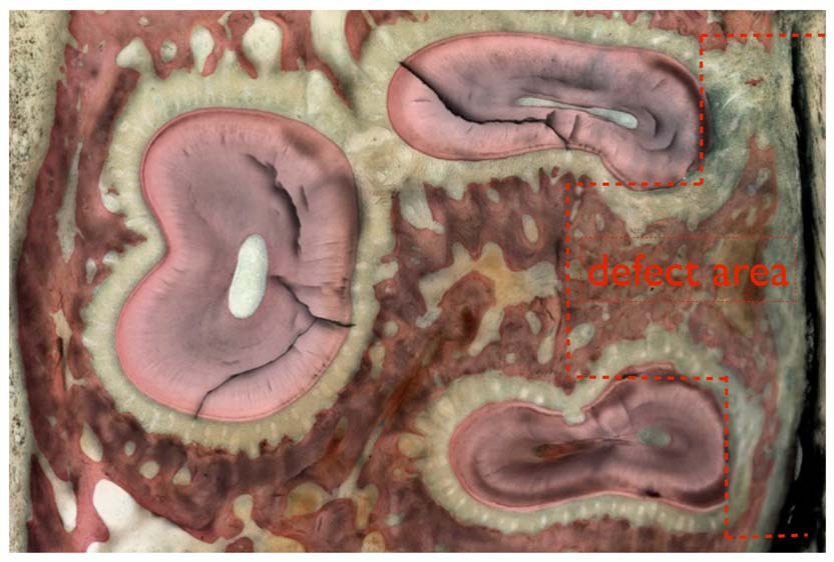
A descriptive histologic section showing the defect area (marked in red) for the maxillary molar.

**Figure 4:**
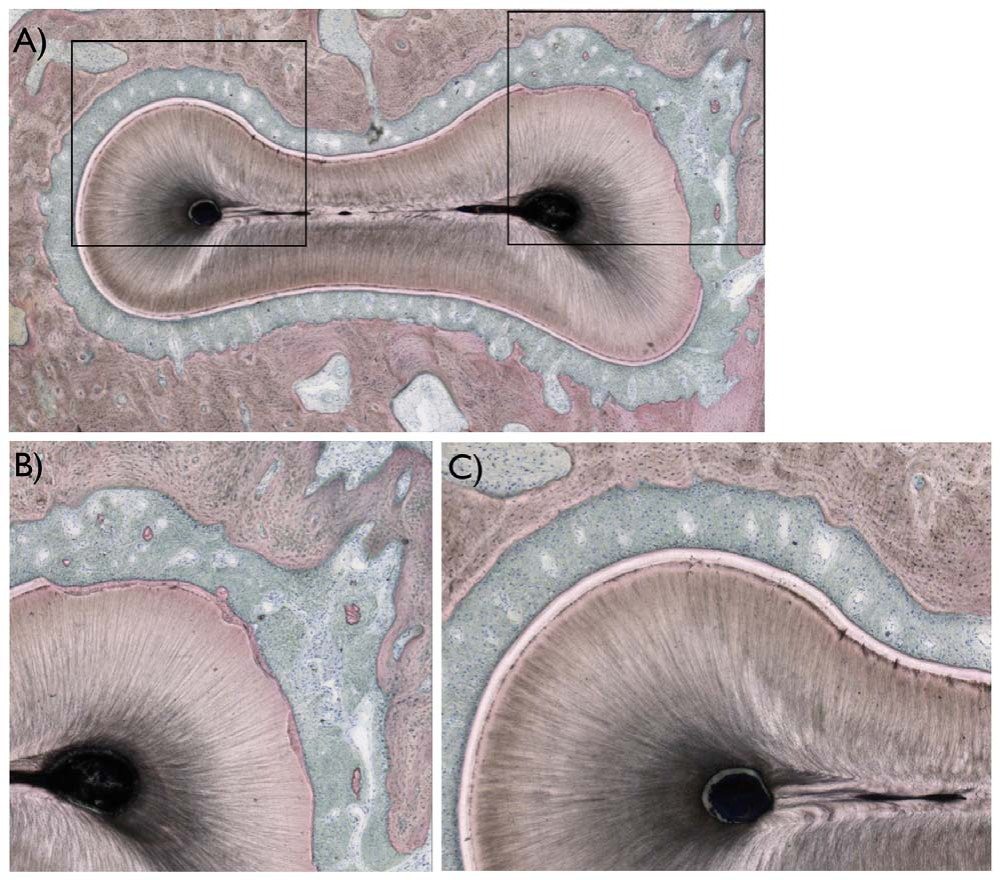
Histologic images of the areas of interest observed in the study. (a) A descriptive histologic section showing the region of interest evaluated (marked in black). (b) For all teeth evaluated, damage to the root was observed primarily at the buccal and furcation region of the roots, where regions of cementum were removed in full thickness and root dentin removed in partial thickness. (c) In regions not surgically affected, the typical anatomic features of hard (alveolar bone, cementum, and root dentin), and soft tissues (periodontal ligament) were observed.

Three histologic sections were obtained for each tooth region operated at both arches, and the sections depicted the region of interest where the defects were created as presented in Figure 3 and 4a. For all sections, surgical damage to the root was observed primarily at the buccal flange and at the furcation region, where the cementum regions were removed in full thickness and root dentin was removed in partial thickness (Figure 4b). In regions not surgically affected, the typical anatomic features of the periodontal tissues were observed (Figure 4c).

No noticeable difference in healing characteristics and inflammatory cell content was observed when sections that originated from the induced inflammation sites were compared to the non-inflamed sites, and thus healing pattern qualification was carried out by collapsing inflamed and non-inflamed sites.

**Figure 5:**
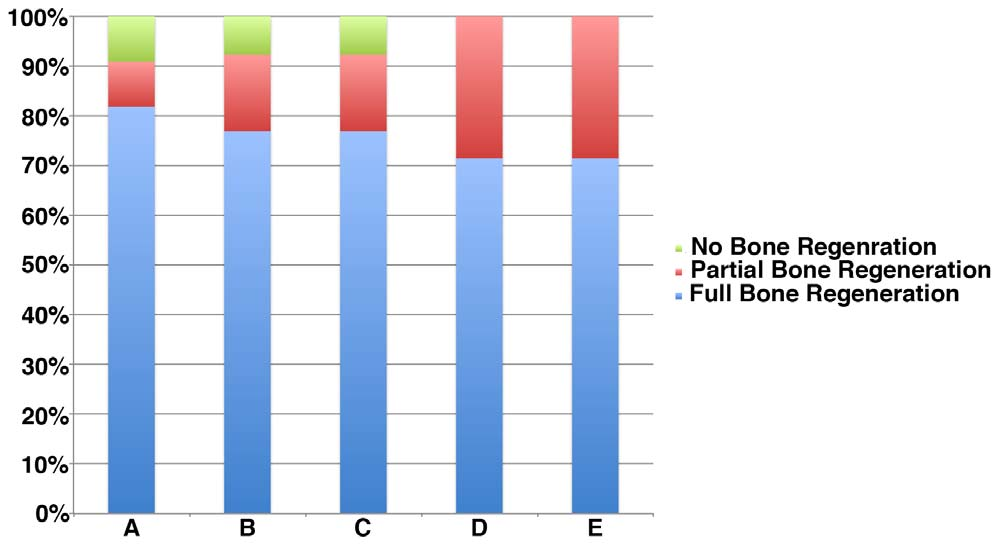
Percent distribution of bone healing pattern, where majority of the samples presented full bone regeneration at the defect region, a smaller fraction partial regeneration, and an even smaller fraction of all samples of the study did not present full buccal bone regeneration.

**Figure 6:**
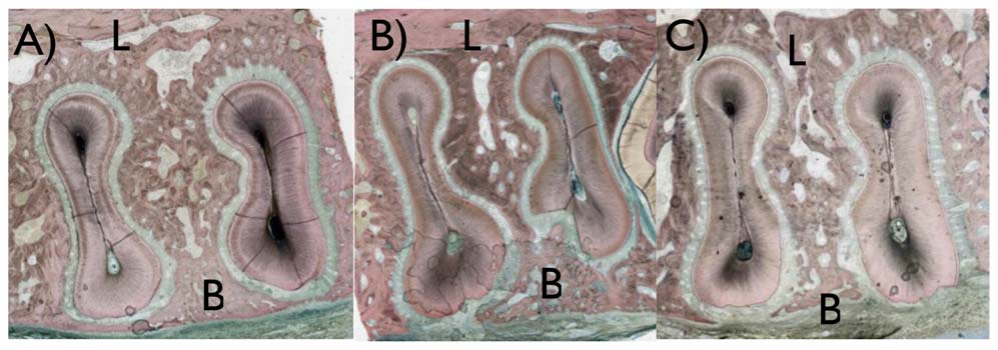
Histologic sections showing the buccal (B) and lingual (L) regions of samples presenting different bone healing patterns. Most of the sites evaluated presented (a) full bone regeneration, and few presented (b) partial regeneration or (c) no regeneration.

The histologic evaluation showed that most (80%) of the sites evaluated presented full regeneration of the bone region within the defect (Figure 5), and few presented partial or no regeneration (Figure 6a-c). Statistical analysis with GEE showed that inflammation led to a lesser likelihood of full bone regeneration (71 vs. 87%) (p = 0.02). There were no statistical differences among experimental groups (p = .95).

**Figure 7:**
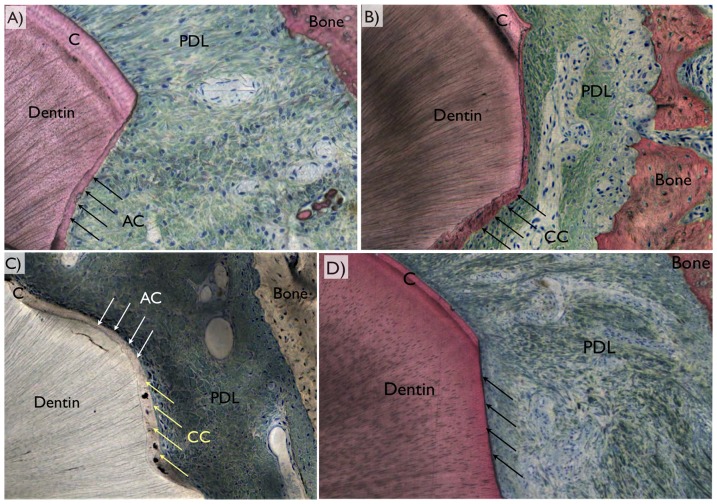
Representative histologic images depicting the periodontal ligament (PDL) region between bone and the tooth, where original cementum thickness (C) could be depicted in the vicinity of a defect. The three different regeneration patterns comprised (a) acellular cementum (AC), (b) cellular cementum (CC), (c) mixed cellular and acellular cementum (MCA), or (d) no cementum regeneration (arrows).

In the cement layer, there was full regeneration in about 40% of the samples. Induced inflammation had little effect on regeneration of this layer (p = .5) but treatment did (p = .04). Post-hoc comparisons indicated the highest level of regeneration, 85% in group A, higher than all other groups (p<.05). Intermediate was group C at 47%, and greater than group D at 14% (p<.05). Groups B and E, 37 and 28%, respectively, were statistically similar to both C and D. Cellular, acellular, mixed cellular and acellular were present in all groups. A remarkable observation was that group C presented primarily acellular cement compared to other groups. Histologic representative samples revealing the PDL region between bone and the tooth, where original cementum thickness in the vicinity of regenerated cementum different morphologies are presented in Figure 7.

In the PDL layer, none of the slices from group D showed full regeneration, and they were omitted from subsequent statistical analysis (the GEE procedure cannot fit a model in the presence of null cells because it requires a division by zero). Among the remaining slices, about 33% showed full regeneration. Inflammation did not affect these values (p = .55), but treatment did (p<.001). Table 1 shows an estimated level, 69%, in group A that was higher than any other group (p<.05). The next highest value, 42% in group E, was greater than group B (12%; p<.05) but similar to group C, at 20%. Group D, with no instances of full regeneration, was statistically lower than any other treatment, as the lower limit in each of those groups did not include zero.

**Table 1:** Estimated mean and 95% confidence interval for full periodontal ligament regeneration.

Estimates				
			95% Wald Confidence Interval	
Group	Mean	Std. Error	Lower	Upper
A	.69	.109	.45	.86
B	.12	.081	.03	.38
C	.20	.085	.08	.42
E	.42	.150	.18	.71

**Figure 8:**
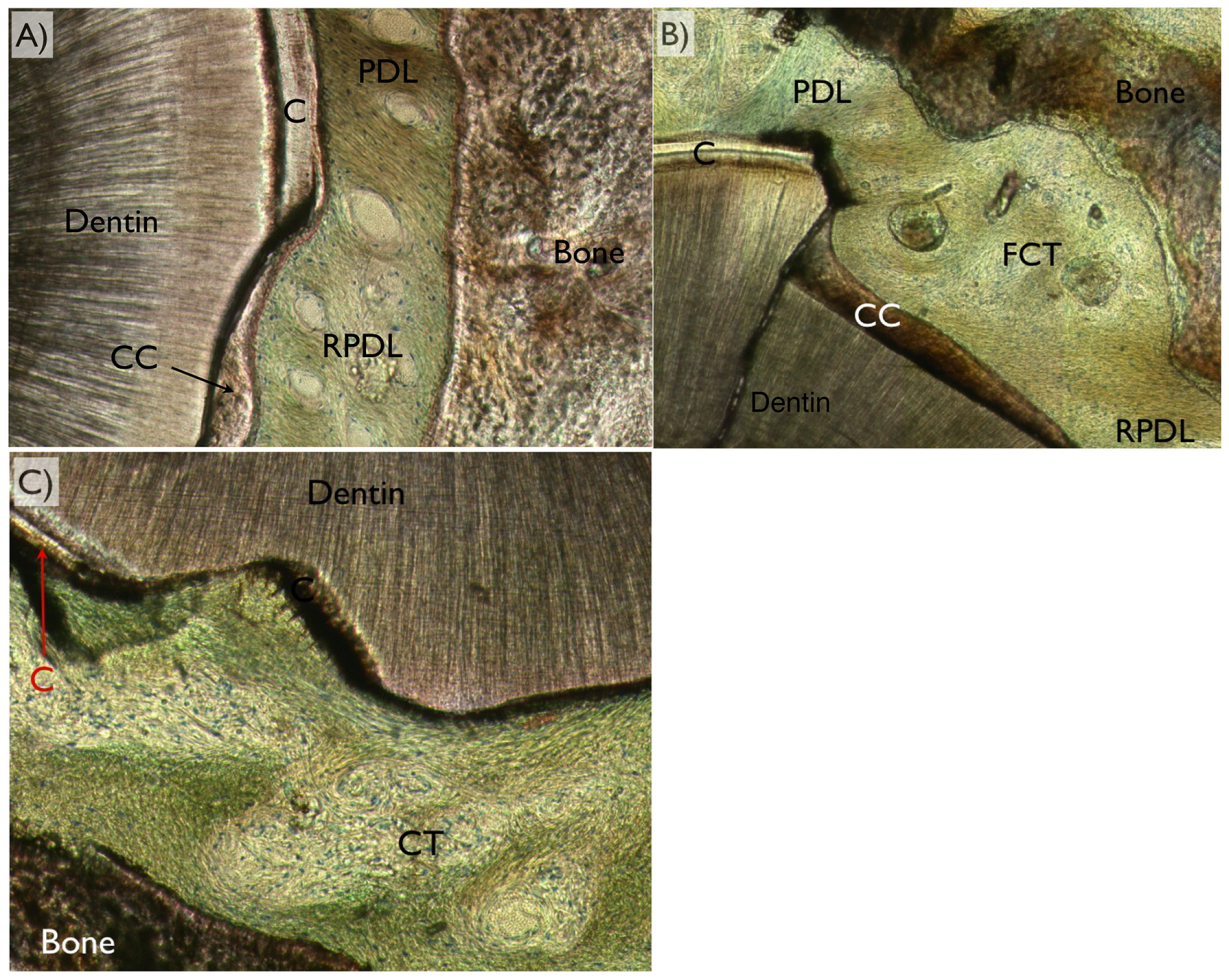
For periodontal ligament regeneration assessment, optical microscopy in circular polarized mode was utilized and the different tissues (dentin, bone, cementum [C], cellular cementum [CC], acellular cementum [AC], original periodontal ligament [PDL], regenerated periodontal ligament [RPDL], and fibrous connective tissue [FCT were easily depicted). Among the three variations of healing pattern observed were (a) full regeneration, (b) partial regeneration, and (c) no regeneration (no fibrous bridging between bone and cementum along the defect perimeter) were observed.

The optical microscopy using circular polarization revealed different tissues (Figure 8). Among the three variations of healing pattern observed were PDL full regeneration, partial regeneration, and no regeneration. It was noticed that full regeneration only occurred when full cementum coverage on surgically removed dentin occurred. Also, partial cementum coverage did not necessarily result in partial PDL regeneration. It was also noted that while defects were fully covered by acellular cement, such condition did not necessarily lead to full PDL regeneration.

**Figure 9:**
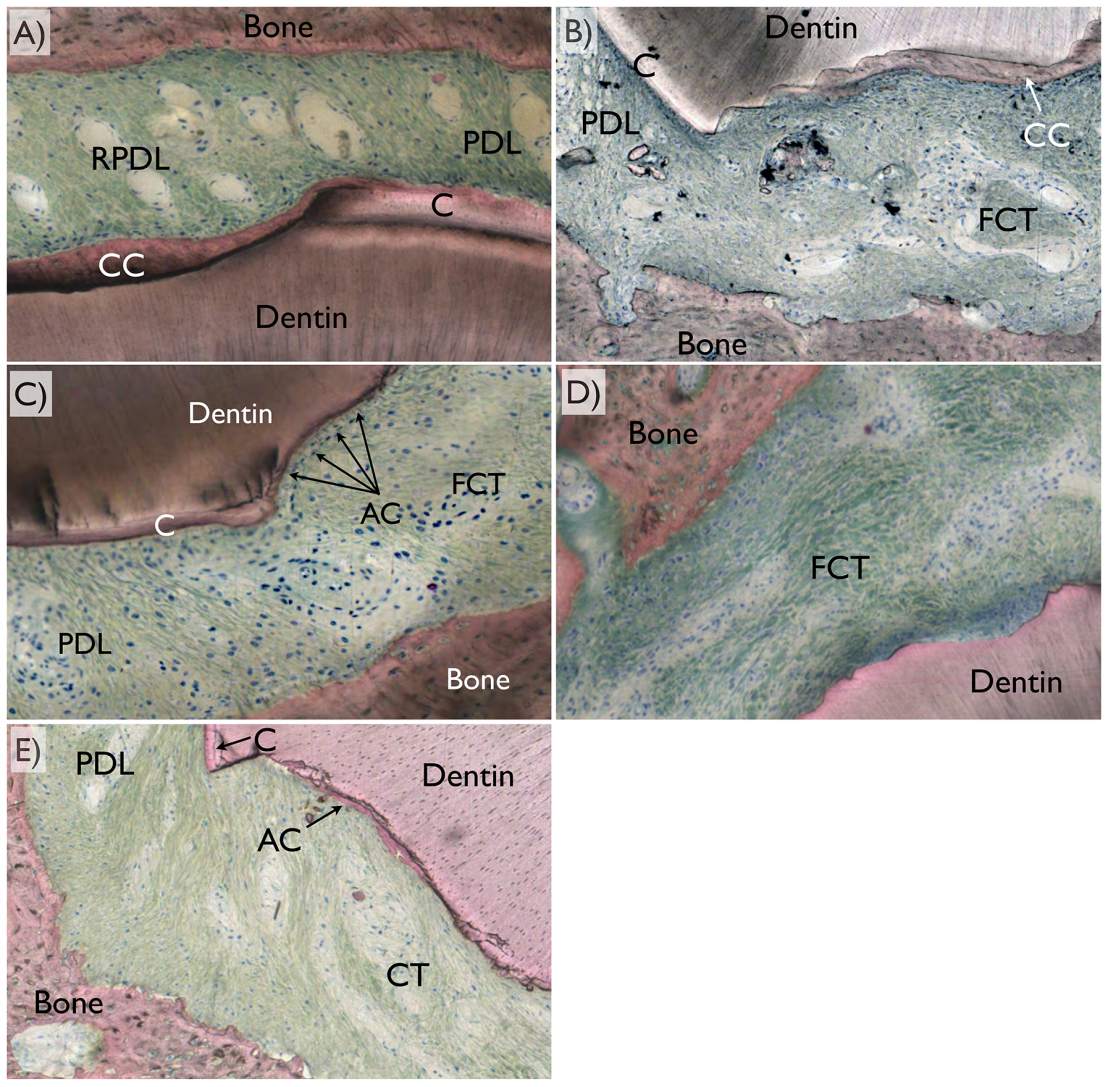
Representative histologic images depicting frequent healing patterns for each group depicting the region comprising bone, periodontal ligament region (PDL), and cementum (C). (a) Group A primarily presented full bone and full defect perimeter cementum regeneration (cellular [CC] and full thickness repair), along with full periodontal ligament regeneration (RPDL) with fibers bridging the gap between the cellular cementum and newly formed bone. (b) Group B primarily presented primarily full bone regeneration along with partial cementum coverage (mostly celular at covered regions) and partial periodontal ligament regeneration. (c) Group C presented full bone and cementum regeneration (partial thickness, acellular) along with partial periodontal ligament regeneration. (d) Group D presented full bone regeneration with partial (mostly acellular) or no cementum regeneration, along with partial or no periodontal ligament regeneration. (e) Group E presented full bone regeneration with partial (mostly acellular) or no cementum regeneration, along with partial or no periodontal ligament regeneration.

The representative samples depicting frequent healing patterns for each group are presented in Figure 9. Group A primarily presented full bone and full defect perimeter cementum regeneration, along with full PDL regeneration with fibers bridging the gap between the cellular cementum and newly formed bone (Figure 9a). Group B primarily presented full bone regeneration along with partial cementum coverage and partial PDL regeneration (Figure 9b). Group C presented full bone and intermediate levels of full and partial cementum along with partial PDL regeneration (Figure 9c). Group D presented full bone regeneration with partial or no cementum regeneration, along with partial or no PDL regeneration (Figure 9d). Group E presented full bone regeneration with partial or no cementum or partial regeneration, along with partial or no PDL regeneration (Figure 9e).

## Discussion

This study tested the hypothesis that BDNF in combination with a synthetic scaffold regenerates the periodontal tissue defect. The term ‘regeneration’ or ‘regenerative’ was used in this study to point out that the intention of the study was to repair as much as possible, the periodontal tissue to its original structure and morphology. Moreover, the alginate material was used to induce an initial inflammatory response, although it must be noted that the induced inflammation referred to in the current study is different from the inflammation caused by periodontal diseases.

Periodontal disease is one of the commonly observed reasons for the destruction of the periodontal tissue, which in the worst scenario may result in the loss of tooth structure [15], [16]. Due to the extensive research in the field, periodontal treatment has now succeeded in preventing the recurrence of the disease [17], [18] and there has been a paradigm shift in the field from halting the progression, to regenerating the once lost tissue by using surgical techniques such as guided tissue regeneration (GTR) with or without the combination with various growth factors [19]–[22].

One of the challenging regenerative sites is the furcation defect. It has been suggested that class II furcation defects can be regenerated with the aforementioned techniques to a certain extent [23], [24]. However, according to Pontoriero *et al.* (1992), the outcomes of such regenerative procedures when applied to class III furcation defects (through and through) were inefficient and was suggested that the application of the GTR technique may not be a predictable solution [25]. On the other hand, it has been reported in *in vivo* canine class III furcation defect studies that the use of BDNF in combination with atelocollagen sponge or with synthesized high molecular weight hyaluronic acid seemed to regenerate the periodontal tissue in a dose dependent manner [10], [14]. Thus, it was of great interest to observe histologically in detail the effect of BDNF in combination with synthetic scaffolds in higher hierarchy animals aimed to correlate to clinical reality.

Class II furcation defect was selected in this study since this defect to a certain extent has been known to regenerate at least the alveolar bone if appropriate space making was granted. In fact, both histologic and histomorphometric outcomes of the current study presented full or partial bone regeneration for all groups and there seemed to be no differences. Therefore, our interest using this setup was to observe and to compare in detail, the differences in the regenerated (complete or partial) periodontal tissues (especially the cementum and the periodontal ligament) between different groups and also between inflammation-induced and non-inflamed sites. Furthermore the study aimed to evaluate the synergistic effect of the BDNF and hyaluronic acid. Although slightly lower degrees of full bone regeneration were detected on induced inflammation sites, besides specific interactions with certain times in vivo and arch, general results showed that the sites subjected to early-induced inflammation healed as well as the non-inflamed sites when alveolar bone, cementum and periodontal ligament regeneration were concerned. Thus, the effect of inflammation was collapsed in the current study when further statistical analyses were performed.

The results presented different regenerative properties for all groups tested with the Group A (BDNF at 500 µg/ml in hyaluronic acid) presenting the most effective outcomes in terms of complete regeneration of the periodontal tissue. Higher dosage of BDNF in hyaluronic acid resulted in the highest degrees of full cementum regeneration (with almost no samples presenting exposed dentin), this being primarily cellular cementum coverage, presumably providing an appropriate environment for periodontal ligament fibers to attach and organize in a fashion similar to periodontal ligament regions which were not affected by the surgical technique.

While the other groups presented partial or full bone regeneration, histologic observations evidenced that the multiple periodontal tissue components did not completely regenerate. Based on the histomorphometric results, it is indicative that full cementum coverage (cellular or acellular) is one of the essential factors for periodontal ligament regeneration. This is supported by Group A having the highest percentage of complete periodontal ligament regeneration.

Interestingly, Group C (hyaluronic acid without BDNF) presented histologically, partial cementum regeneration, however, the main constituent was acellular, and compared to Group A, this group did not result in high degrees of periodontal ligament regeneration with appropriate attachment of fibers in the regenerated cementum and bone. Hyaluronic acid is a connective tissue glycosaminoglycan that enhances would healing, cell migration, and differentiation during tissue repair [26], [27]. It has been utilized for the reinforcement of periodontal ligament sheets to compensate for their fragile property and has been shown to be effective in accelerating the wound healing process [28]. However, compared to the group presenting high BDNF dosage in hyaluronic acid, it possibly did not have the specific signaling pathway to influence the differentiation of the cementum. Therefore, this may be one of the reasons why the cementum and periodontal ligament regeneration was incomplete for this group. Nonetheless, the utilization of hyaluronic acid carrier alone presented better overall results than all other groups but group A (high BDNF dosage in the hyaluronic acid carrier).

It is of note that when the lower BDNF dosage was used (Group B), or BDNF alone in saline solution was employed (Group E), results were no different than the control Group D (no material placement in defect), where lower levels of regeneration were observed for both cementum and periodontal ligament. This may be that lower BDNF was not effective to fully regenerate the periodontal tissue, since the effect of BDNF is again, solely dose dependent [10], [29]. Further, without the presence of a proper carrier, the effect of BDNF cannot be exerted and be altered by other growth factors existing in the bodily fluid.

To conclude, the brain derived neurotrophic factor (BDNF), in combination with a synthetic hyaluronic acid carrier induced higher degrees of complete periodontal tissue healing in a dose dependent manner supporting the postulated hypothesis. It is strongly suggested that the application of this neurotrophin-carrier complex in a periodontal tissue defects may result in its nearly complete regeneration.
